# ‘They have a right to participate as a stakeholder’: Article 5.3 implementation and government interactions with the tobacco industry in Ethiopia

**DOI:** 10.1136/tobaccocontrol-2021-056885

**Published:** 2022-01-31

**Authors:** Selamawit Hirpa, Rob Ralston, Wakgari Deressa, Jeff Collin

**Affiliations:** 1 Department of Preventive Medicine, School of Public Health, Addis Ababa University, Addis Ababa, Ethiopia; 2 Global Health Policy Unit, Social Policy, School of Social and Political Science, University of Edinbrugh, Edinburgh, UK

**Keywords:** tobacco industry, public policy, global health

## Abstract

**Introduction:**

This paper explores implementation of Article 5.3 of the WHO Framework Convention on Tobacco Control in Ethiopia. The analysis highlights how operationalising key requirements of Article 5.3, such as minimising policy engagement with the tobacco industry, has been mediated by path-dependent processes of stakeholder consultation and collaboration that have persisted following privatisation of Ethiopia’s state-owned tobacco monopoly.

**Methods:**

We conducted semistructured interviews with key officials (n=21) working in tobacco control policy, with high levels of access secured across ministries and agencies beyond health.

**Results:**

We found contrasting levels of awareness of Article 5.3 across government sectors, with extremely limited awareness in departments beyond health. The data also highlight competing ideas about conflict of interest, in which recognition of a fundamental conflict of interest with the tobacco industry is largely confined to health actors. Beyond limited cross-sectoral awareness and understanding of Article 5.3, gaps in implementation are exacerbated by assumptions and practices around stakeholder consultation, in which attempts to minimise policy interactions with the tobacco industry are mediated by institutionally embedded processes that presume active engagement. The results also highlight a continuation of the close relationship between the Ethiopian government and tobacco monopoly following its privatisation.

**Conclusion:**

The Ethiopian government’s recent achievements in tobacco control legislation require that policymakers are actively supported in reconciling perceived tensions and requirements for stakeholder consultation. Effective tobacco control governance would be further enhanced by enabling government agencies to more clearly identify which interactions with the tobacco industry are strictly necessary under Article 5.3 guideline recommendations.

## Introduction

International recognition that inadequate protection against tobacco industry interference is the key barrier to effective implementation of the WHO Framework Convention on Tobacco Control (FCTC) extends across tobacco control experts, advocates and governments.^1–4^ Despite the strategic significance attached to Article 5.3 and its associated guidelines requiring parties to protect tobacco control policy from industry interests, its implementation has generally been weak^5^ and there is a dearth of detailed qualitative analyses of the policy challenges of tobacco control governance confronting policymakers and officials. While such research in high-income country contexts has been limited,^6 7^ the absence of such studies in regions confronting the most serious challenges is striking. This is particularly the case in sub-Saharan Africa, despite the rapid expansion of the tobacco industry across the region.^8 9^ As Africa’s second most populous country, Ethiopia has emerged as a key market for industry expansion,^10 11^ marked by the privatisation of its state-owned tobacco monopoly. Starting with a 30% share in the monopoly bought in 1998 by a Yemeni investment company,^12^ the National Tobacco Enterprise (NTE) has transferred to Japan Tobacco International (JTI), which recently acquired a controlling stake, an initial $510 million acquisition of 40% in 2016 and an additional 30% of total shares for $434 million in 2017.^13^ Announcing its initial investment in NTE, JTI asserted, “Ethiopia will be an important expansion of our geographic footprint in emerging markets”, citing ‘double-digit economic growth’ and expectations that volumes would continue to rise.^14^ While Ethiopia has comparatively low rates of tobacco smoking in the region,^15^ retail sales of tobacco have steadily increased post-privatisation, with industry analysts reporting rising sales among young adults in urban areas and increased social acceptability of cigarettes among women.^16 17^ JTI is positioned to exploit economic and population growth with a monopoly on tobacco imports and export until 2025, introducing its *Winston* brand in 2019 and distributing other leading international brands such as *Marlboro* and *Rothmans*.^16^

Ethiopia constitutes an important context in which to examine efforts to implement Article 5.3, its distinctiveness being reflected in negotiations between the Ethiopian government and JTI over privatisation concluding with significant efforts by officials and advocates to advance tobacco control legislation. While earlier tobacco regulations primarily served to protect the interests of the monopoly,^18^ a National Tobacco Control Directive was introduced following FCTC ratification in 2014. This aimed to advance implementation of measures including Article 6 on taxation and Article 11 on packaging and labelling, but notably omitted any provisions on regulating tobacco industry interference.^19^ In 2019, primary legislation (known as Proclamations) was unanimously passed by the Ethiopian House of People’s Representatives to enact one of the most comprehensive laws in Africa.^20^ This Proclamation (1112/2019) signals a step change in tobacco control governance, including in seeking to implement key elements of Article 5.3 implementation guidelines.^1^ The codification of practices to limit government–industry interactions to those ‘strictly necessary for […] effective regulation’^21^ commits the Ethiopian government to an active policy of minimising policy engagement with it.^22^ Yet, its incorporation of Article 5.3 guidelines is partial and inconsistent (see table 1). Noticeably absent in the Proclamation are guideline recommendations that reject partnership and non-binding agreements with the tobacco industry; do not give preferential treatment to the tobacco industry and treat state-owned tobacco in the same way as any other tobacco industry.^23^

**Table 1:** A comparison of guidelines to limit industry interference in public policy

WHO guidelines for implementation of FCTC Article 5.3	Proclamation 1112/2019, Ethiopia	Extent of fit
Raise awareness about the addictive and harmful nature of tobacco products and about tobacco industry interference with parties’ tobacco control policies		Omitted completely
Establish measures to limit interactions with the tobacco industry and ensure the transparency of those interactions that occur	Interactions between any government organ responsible for the adoption of public health policy and the tobacco industry shall be limited to only those strictly necessary or effective regulation of the tobacco industry or tobacco products Any interaction made in accordance with subarticle (1) of this article, and whenever the tobacco industry contacts the government to initiate an interaction of any kind, the appropriate government official shall ensure full transparency of the interaction and of the contact, and it shall be appropriately documented	Broad consistency
Reject partnerships and non-binding or non-enforceable agreements with the tobacco industry		Omitted completely
Avoid conflicts of interest for government officials and employees	No person having financial or other interest in the tobacco industry may participate in tobacco control training, workshop or related events unless in accordance with an invitation by the relevant health regulator No government organ or an official working in the area of health policy should receive any financial or in-kind contribution from the tobacco industry	Broad consistency
Require that information provided by the tobacco industry be transparent and accurate		Omitted completely
Denormalise and, to the extent possible, regulate activities described as ‘socially responsible’ by the tobacco industry, including but not limited to activities described as ‘corporate social responsibility’	Any financial or in-kind charitable or any other related contribution by a tobacco industry shall be prohibited	Partial coverage
Do not give preferential treatment to the tobacco industry		Omitted completely
Treat state-owned tobacco industry in the same way as any other tobacco industry		Omitted completely

FCTC, WHO Framework Convention on Tobacco Control.

The omission of Article 5.3 guidelines to reject partnership with the tobacco industry occurred in the context of a Memorandum of Understanding (MoU) between the Ethiopian government and JTI that creates a ‘framework of cooperation between the parties’ to address illicit tobacco trade.^24^ This non-binding voluntary agreement establishes collaboration between NTE (represented by JTI officials) and the Ethiopian Customs Commission including training and information sharing. These developments in Ethiopia’s tobacco industry have shaped the institutional and political context in which legislation to minimise interactions with the tobacco industry is being operationalised.

This paper seeks to examine the challenges involved in implementing Article 5.3 amid such complex and countervailing pressures, analysing four key themes arising from interviews with officials working across the Ethiopian government: (1) contrasting levels of Article 5.3 awareness across ministries; (2) details competing perceptions of conflict of interest with the tobacco industry; (3) explores tensions with the Ethiopian government’s wider commitments to stakeholder consultation in policy development and (4) the legacy of state ownership of the tobacco monopoly in institutionalising ongoing collaboration.

## Methods

The analysis is based on 21 interviews with key officials, civil servants and policymakers from across sectors, with high levels of access secured to actors within government agencies and departments beyond health (table 2). Officials in ministries of finance, trade and revenue and customs authority were selected based on their departmental roles in FCTC implementation post-privatisation. This approach ensured that diverse perspectives and experiences were captured in the data, with interviewees varying in their proximity to tobacco control policymaking, awareness of Article 5.3 and interactions with the tobacco industry. Similar diversity applied to disciplinary and professional roles, with interviewees including legal consultants, economists, health workers, researchers and policy specialists.

**Table 2:** An overview of interviewees by sector

**Health departments**	**10**
Ethiopian Food and Drug Administration	
Ministry of Health	
Regional health agency	
Government health research institution	
**Departments beyond health**	**7**
Ethiopian Revenues and Customs Authority	
Cabinet Office	
Elected official	
Ministry of Finance	
Ministry of Trade	
Non-governmental organisation and health advocates	4

Interviews were semistructured, employing an interview schedule covering awareness of FCTC Article 5.3 and its guideline recommendations; Article 5.3 implementation; approaches to interaction or collaboration between government and the tobacco industry. The semistructured approach enabled the interview schedule to be adapted according to the departmental role of policy officials, and included questions relating to Proclamation 1112/2019, privatisation of the state-owned tobacco monopoly, and interactions between JTI and the Ethiopian government. In addition to the interview schedule, printed copies of WHO Article 5.3 guidelines were taken to interviews, as both an aide-mémoire for interviewers and performing a more interactive role in interviews where policy officials had limited awareness of Article 5.3 or of Proclamation 1112/2019.

In presenting these data, we distinguish between policymakers with professional roles and responsibilities within the Ministry of Health and Ethiopian Food and Drug Administration (EFDA), and interviewees in government departments and agencies beyond health (table 2). This aims to balance clarity about institutional affiliation and context with both protecting anonymity and political sensitivities.

Interviewees were identified by SH and contacted via telephone, with interviews conducted by researchers at the School of Public Health, Addis Ababa University. Interviews varied between 25 and 75 min (with most around 40 min) and were conducted in a private space where only the interviewee and researcher were present. Interviewees were asked to sign a consent form that allowed interviews to be digitally recorded and for the data to be used in research outputs. All interviews were conducted in Amharic, transcribed and anonymised, before translation into English. Interview transcripts were analysed in NVivo using a thematic coding framework which was developed iteratively through descriptive and then conceptual coding. This started with descriptive codes relating to experiences and awareness of policymaking practices, contextualised with key concepts developed within policy studies and political sciences. This included a particular focus on the concept of path dependence, referring to the tendency for persistence in institutional practices and policies, as ‘once particular paths have been forged, it requires a significant effort to divert them on to another course.’^25^ The analytical approach was iterative, with interview transcripts analysed and re-analysed to identify different perceptions of tobacco control governance. Interview data were complemented by a review of policy and legal documents identified by interviewees. These include a 2004 *Minister of Council’s Implementation Directive* 26 that establishes formal procedures for stakeholder consultation in developing legislation, in addition to an MoU on illicit trade negotiated between the Ethiopian government and NTE/JTI in 2019.

All interview transcripts were coded by SH and RR with input from JC. The initial findings were reviewed at a Global Challenges Research Fund consortium meeting in Addis Ababa, Ethiopia in February 2020, and developed via coordination calls between SH, RR and JC.

## Results

### Contrasting levels of Article 5.3 awareness

A notable aspect of the data was the contrasting levels of awareness of Article 5.3 across agencies, with familiarity of this policy instrument restricted to policy officials in health departments. Several interviewees openly stated that they were not aware of Article 5.3 alongside a lack of in-depth knowledge of tobacco control laws. As one interviewee from a non-health department put it, “I don’t know the Article [and] only know a little about the new Proclamation.”

This contrasted with policymakers in EFDA (the executive agency with formal responsibility for tobacco control), with several interviewees detailing the purpose of Article 5.3 and the norm of a fundamental conflict between tobacco industry and public health interests that underpins its guideline recommendations. As one such interviewee noted, “If we look at Article 5.3, it is about tobacco industry interference […] one of the guiding principles is that there is a conflict of interest between public health policy and the tobacco industry.” Interviewees also pointed to the codification of Article 5.3 in tobacco control law, highlighting that:

Article 5.3 is about the relationship between the tobacco industry and government institutions. In the new Proclamation, we have added an article concerning communication between government and the tobacco industry which has 3 or 4 recommendations […] so Article 5.3 is included in the new law.

While interviewees from EFDA (all of which had direct responsibilities in tobacco control) demonstrated a high level of knowledge about Article 5.3 and the recent inclusion of key guideline recommendations in law, several (in addition to a Cabinet Office official) raised doubts about levels of awareness in other government sectors. One EFDA policymaker acknowledged, “That the Proclamation and Framework [Convention] need to be reconciled. Nobody is aware of [Article 5.3] and currently [the tobacco industry] can collaborate with government institutions and this will be incentivised because of the jobs the industry creates.” There was a consensus among such interviewees that Article 5.3 guidelines had to become embedded across government sectors, with one noting: “We need to work on creating awareness that institutions should not have any partnership with the tobacco industry. Most institutions may not know that they should not work [with the tobacco industry]. I can’t say that everyone knows about the FCTC—it’s only those working in [tobacco control] that have this knowledge.”

There were broad concerns that limited awareness of Article 5.3 increased the likelihood that other government agencies would endorse, support or form partnerships with the tobacco industry. As one interviewee acknowledged:

The FCTC states that there should be no partnership between the government and industry […] but a lot has to be done horizontally across [government]. As I said to you previously, that is why JTI have an agreement on illicit trade with Customs. The legal framework is strong, but Article 5.3 implementation should be cross-government. It should not only be a concern for the Ministry of Health. All sectors like finance, trade, customs and revenue are bound to the law.

### Competing ideas about conflict of interest

While EFDA interviewees consistently identified a fundamental conflict of interest between the tobacco industry and public policy, individuals in other government sectors tended to convey the desirability of policy engagement with industry; this was epitomised by the suggestion, “it would be good for [policy makers] like me to get along well and discuss policy” with industry. In particular, several interviewees from the revenue and customs authority identified illicit trade as an issue in which JTI interests closely aligned with those of the Ethiopian government. One such official claimed, “There is a mutual interest in preventing illegal trade. It is in the interests of [government] and industry. It is a mutual interest; there are no conflicting ideas. The government needs to work in coordination in the area of [tobacco] smuggling to get the benefits of the tobacco sector.”

Echoing principles underpinning Article 5.3, EFDA interviewees were overtly critical of the idea that industry interests could be so aligned with public health, remarking, “There is a definitely a conflict of interest” and “our goals are totally opposite […] the tobacco industry is not our partner. We have different and opposing goals.” Yet, there was some variation among health officials in attitudes towards policy engagement with the tobacco industry. One health official argued, “It is good to work closely […] when we mean conflict of interest, it doesn’t mean to never meet” while another justified consultation on the basis that “the law is implemented on their end, so there should be participation on some issues […] persuading them is important.”

### Requirements for stakeholder consultation and interactions with the tobacco industry

The perception that policy engagement with the tobacco industry was often legitimate appeared consistent with well-established requirements for stakeholder consultation in Ethiopia. A 2004 directive^26^ issued by the Council of Ministers (the Cabinet of Ethiopia) requires that ‘all opinions are reflected’ in policy and legislative decision-making, including provision that ‘if found necessary, there may be consultation with organised groups during the process of preparing draft policies’ (box 1). While Article 5.3 guidelines allow restricted tobacco industry consultation to take place as long as it is transparent and accountable,^23^ the interview data highlight that this requirement has been interpreted by officials within the Ethiopian government as reinforcing practices of dialogue and policy coordination with the tobacco industry.

Box 1 **Minister of Council’s Implementation Directive (2004)**

**Procedural steps on drafting policy and legislation**

*Procedures on drafting policy*

31. Consultation with concerned government bodies.

31.04 The minister who originated the draft should make sure sufficient consultations are held between different parties and all opinions are reflected. This helps to avoid any disagreements and facilitates the draft document’s approval at the council’s meeting.

32. Consultation with organised groups.

32.1 If found necessary, there may be consultation with organised groups during the process of preparing draft policies.

32.3 If there is a proclamation requiring the need for consultation before implementation of the law, the minister should make sure sufficient consultations are conducted as per the requirements.

Despite Proclamation 1112/2019’s incorporation of Article 5.3 guidelines to limit policy engagement with the tobacco industry, several interviewees highlighted how this principle came into conflict with stakeholder consultation practices. Interviewees from both EFDA and the Cabinet Office described how industry participation in policy development has remained largely unchanged, following the strengthening of tobacco control law:

When a law is drafted, they have the right to participate as a stakeholder. We have to comply with that requirement and engage with them before [tobacco control] laws are approved […] the requirement of the Council of Minister’s directive [is that stakeholders] need to be engaged and give their opinion before approval […] if we didn’t engage them [the law] would have been delayed and [the Council of Ministers] will say that you need to engage stakeholders.

[…] when you prepare a draft law, you have to involve all stakeholders. In this case that means the tobacco industry, organisations that work on health issues, Customs and the Ministry of Finance. You have to engage them all […] what we do is make sure they [tobacco industry] are involved during meetings that are held at ministry level.

This suggests that implementation of Article 5.3 in Ethiopia is being significantly shaped by institutionalised procedures of stakeholder consultation, and both interviews saw this as being in tension with FCTC norms. Another FDA interviewee sought to reconcile these procedural constraints as compatible with commitments to prevent tobacco industry interference, reflecting, “The Ministry of Council’s requires that all stakeholders to participate. So, we invite the tobacco industry to comply with this requirement. But that does not mean we allow them to interfere—we stand to protect public health.”

For interviewees from the Revenue and Customs Authority, consulting the tobacco industry was viewed as signalling a commitment to transparent and accountable governance. As one senior policymaker described, “When you make a Proclamation there is something to talk about […] it will be presented for consultation and [the tobacco industry] will have their say. There will be situations where they can request adjustments.”

The notion of the tobacco industry as a legitimate stakeholder was also strikingly articulated by an EFDA interviewee, who suggested that:

The law will [impact] on the tobacco industry; that means they are one of the stakeholders. Anybody who is going to implement [policy] should discuss their concerns before you have approved that law. We need to hear what they say concerning the law and we need to explain to them how they are going to implement it, and if they have any reasonable points we need to entertain them.

This view diverged sharply from all other EFDA interviewees, with one senior policymaker reflecting on discomfort and uncertainty about how to manage competing expectations: “[T]here was a discussion about why we need to engage the tobacco industry […] because public health and industry interests are irreconcilable, they should not be engaged. On the other hand, if we know we should not engage them, it is a requirement of [the directive] to engage stakeholders. So, to not delay the approval of laws we need to engage. This is why we need to engage them.” This perceived tension between stakeholder consultation and Article 5.3 implementation was captured by one interviewee:

There is a lack of clarity on this. The [FCTC] prohibits them to get involved or influence [policy] processes due to a conflict of interest, but our country’s law dictates involvement of all stakeholders during the preparation of any draft law.

### The political legacy of state ownership: partnering with JTI on illicit tobacco trade

Alongside tensions between codified Article 5.3 guidelines and wider governance practices, inconsistent and partial approaches to limiting interaction with the tobacco industry also appear closely linked to the privatisation of NTE and evolving relationship between JTI and the Ethiopian government. Several interviewees highlighted the historically close relationship between the Ethiopian government and NTE, with one policymaker reflecting that board members ‘were assigned by government’ and ‘accountable to the ministry [of public enterprise].’ These organisational ties were also cited by one senior official interviewee, who suggested, retrospectively, that the interests of Ethiopian government and NTE were closely linked.

While privatisation has modified the government’s responsibilities and interests in the tobacco industry, collaboration with NTE has been maintained. This continuity is evident in the relationship between the Ethiopian Revenue and Customs Authority and NTE, with interviewees within the department describing the MoU negotiated with JTI as a mutually beneficial arrangement in addressing illicit tobacco trade. For example:

There is a mutual interest in preventing illegal trade protecting itself from illegal tobacco imports—supporting each other, especially in customs enforcement […] they have the financial capacity, the information […] it is about working together and what you have to collaborate on. The government has to be in a place to cooperate with the private sector, including tobacco.

This sentiment was reiterated by another official, who argued, “In our work the industry has no interference […] if the government and industry work together it will have a positive impact on the control of tobacco.” The negotiation of an MoU, and the collaborative approach to illicit tobacco trade it embeds, has been a source of tension with Proclamation 1112/2019 and the implementation of Article 5.3 guidelines. Several interviewees (including EFDA officials) explained how public health concerns about collaboration with the tobacco industry on illicit trade were minimised in relation to attracting foreign direct investment. As the following interviewee reflected:

There [were] many conditions that were laid when this agreement took place, mainly to protect and privilege them. One of the protections they [JTI] wanted was from illicit products […] their involvement in illicit trade was raised at the time, but their counterargument was ‘if were are to invest in this country, you have to protect us from illicit trade’.

Importantly, this agreement was described by EFDA interviewees as shaping tobacco control governance, with one interviewee claiming, “Cabinet ministers put pressure on us by saying, ‘we have already made these agreements with them’.” This suggests that a close and collaborative relationship with the Ethiopian government has been maintained despite the transition from state-owned tobacco monopoly.

## Discussion

Exploring policymakers’ experiences of Article 5.3 implementation in Ethiopia provides a powerful lens though which to examine the challenges of developing effective tobacco control governance in a complex national policy context. The interview data demonstrate twin impediments to realising Article 5.3’s potential to underpin coherent and coordinated multisectoral tobacco control policies^22^; both awareness of its provisions and recognition of a fundamental conflict of interest with the tobacco industry are largely confined to health actors. Consistent with studies in other country contexts,^1 5 7 27–29^ the findings point to contrasting levels of awareness of Article 5.3 across government sectors. While policymakers interviewed from EFDA (the executive agency with formal responsibility for tobacco control) embodied a high degree of knowledge about Article 5.3 and its principles, awareness of this policy instrument was almost non-existent in departments beyond health. Importantly, the data also highlight competing ideas about conflict of interest, in which recognition of the fundamental tension between tobacco industry and public health interests, identified by EFDA interviewees, was not shared by interviewees working in other government agencies. This was particularly the case for policymakers in the Ethiopian Revenue and Customs Authority, many of whom felt there was a shared interest between government and industry in addressing illicit tobacco trade. This presumption of shared interests as providing scope for collaboration with the tobacco industry constitutes a specific ongoing challenge in the context of the Illicit Trade Protocol.^30–32^

Beyond limited awareness and understanding of Article 5.3 across key government departments, however, gaps in its implementation are further exacerbated by assumptions and practices around stakeholder consultation. The interview data demonstrate how attempts to minimise policy interactions with the tobacco industry are compromised by institutionally embedded processes that presume active engagement. The difficulties policymakers experience in managing such tensions have previously been noted in high-income contexts,^7 33^ as have industry efforts to exploit tools such as impact assessment and the better regulation agenda.^34 35^ Similar issues have arisen in South Africa, where constitutional requirements for consultation led to the tobacco industry litigating to be recognised as a stakeholder.^8 36^ The analysis presented here suggests that in Ethiopia, the institutional context and ideas about interactions with the tobacco industry heighten such challenges. Concerns about a ‘good governance’ trap,^33^ in which tobacco control is undermined by key government agencies viewing consultation with the tobacco industry as routine and necessary, are likely to be particularly acute in Ethiopia. This reflects both the government’s commitment to economic liberalisation^16 17^ and the governance agenda of key donors such as the World Bank and European Union, using aid conditionality to support wider reforms that embed stakeholder consultation.^37–41^

The paper also highlights the continuing relevance of longstanding practices and assumptions about engagement with the NTE. Despite both JTI’s acquisition of the monopoly and significant developments in tobacco control in Ethiopia, there has been substantive continuity in interactions and relationships with key agencies. The ongoing relevance of the legacy of state ownership is reflected in Proclamation 1112/2019 omitting those elements of Article 5.3 implementation guidelines that most directly circumscribe government-industry relations (in requiring rejection of partnership and non-binding agreements; not giving the industry preferential treatment and not favouring state-owned tobacco interests).

The MoU negotiated between the Ethiopian Customs Commission and JTI on illicit tobacco trade provides a striking example of the value to the NTE of maintaining its close and collaborative relationship with the government. This partnership was frequently cited by interviewees to legitimate policy engagement with the tobacco industry, which suggests that institutional contexts have worked to shape ideas and policy frames around conflict of interest. The data suggest high levels of path dependency^25^ post-privatisation, where expectations about how to engage with the JTI-owned company remain defined by norms and practices developed under state ownership. The relevance of these dynamics to tobacco control governance merits more detailed exploration.^12^

Recognition of ongoing challenges to implementation of Article 5.3 should not detract from Ethiopia’s recent achievements in tobacco control and in managing industry interference. Omissions notwithstanding, its legislation fares comparatively well amid the limited extent of international progress.^3^ Realising its potential to support effective tobacco control governance requires actively supporting policymakers in reconciling perceived tensions with requirements for stakeholder consultation. The question of whether corporate actors constitute stakeholders to be routinely engaged in health governance is increasingly contentious,^42^ and unfortunately Article 5.3 guidelines do not directly engage with this terminology. The variable understanding and uncertainty evidently in Ethiopia and elsewhere^8^ highlight the importance of efforts to more clearly delineate legitimate stakeholders in developing tobacco control strategies.^43^ Effective Article 5.3 implementation would be further enhanced by enabling government agencies to more clearly identify which interactions with the tobacco industry constitute the minimum ‘strictly necessary’.^23^ In Ethiopia, there is clear scope to reconcile this with requirements for stakeholder consultation that are explicitly subject to an ‘if found necessary’ provision (1/1987). More effectively managing tobacco industry interference therefore requires better understanding of which interactions with industry are necessary for policymakers. Participatory research to better identify precise terms of restricted engagement on very specific issues is necessary to prevent tobacco control being undermined by a presumed right to or need for wider consultation with the tobacco industry.

**What this paper adds**

- Protection against tobacco industry interference is a key barrier to effective implementation of the WHO Framework Convention on Tobacco Control (FCTC). Despite the importance of Article 5.3 of the WHO FCTC in limiting industry interference, existing research in high-income country contexts has highlighted partial and uneven implementation of this article.
- This study explores implementation of Article 5.3 in Ethiopia, addressing the absence of empirical studies in sub-Saharan Africa.
- The study highlights limited awareness of Article 5.3, competing ideas about conflict of interest, and the extent of presumed shared interests and scope for collaboration with the tobacco industry.
- Challenges of Article 5.3 implementation in Ethiopia are exacerbated by broader processes of stakeholder engagement and by the institutional legacy of state ownership.

## Data Availability

Data sharing not applicable as no datasets generated and/or analysed for this study. The datasets used and analysed for the current study will be available from the corresponding author on reasonable request

## References

[1] Malone RE , Bialous SA . WHO FCTC article 5.3: promise but little progress. Tob Control 2014;23:279–80. 10.1136/tobaccocontrol-2014-051817 24928868

[2] Puska P , Daube M , WHO FCTC Impact Assessment Expert Group . Impact assessment of the who framework convention on tobacco control: introduction, general findings and discussion. Tob Control 2019;28:s81–3. 10.1136/tobaccocontrol-2018-054429 30181384 PMC6589462

[3] Assunta M . Global tobacco industry interference index. Bangkok, Thailand: Global Center for Good Governance in Tobacco Control (GGTC), 2020. https://exposetobacco.org/wp-content/uploads/GlobalTIIIndex2020_Report.pdf

[4] WHO . Global progress report on implementation of the who framework convention on tobacco control. 2018. Geneva: World Health Organization, 2018. https://www.who.int/fctc/reporting/WHO-FCTC-2018_global_progress_report.pdf

[5] Fooks GJ , Smith J , Lee K , et al . Controlling corporate influence in health policy making? an assessment of the implementation of article 5.3 of the world Health organization framework convention on tobacco control. Global Health 2017;13:12. 10.1186/s12992-017-0234-8 28274267 PMC5343400

[6] Willemsen MC , Fooks G . Tobacco industry access to policy elites and the implementation of article 5.3 of the who framework convention on tobacco control. Tobacco Control 2020;29:e50–5. 10.1136/tobaccocontrol-2019-055251 31848311

[7] Hawkins B , Holden C . European Union implementation of article 5.3 of the framework convention on tobacco control. Global Health 2018;14:79. 10.1186/s12992-018-0386-1 30071862 PMC6090908

[8] Wisdom JP , Juma P , Mwagomba B , et al . Influence of the who framework convention on tobacco control on tobacco legislation and policies in sub-Saharan Africa. BMC Public Health 2018;18:954. 10.1186/s12889-018-5827-5 30168395 PMC6117626

[9] Siddiqi K . Tobacco use in sub-Saharan Africa: the risks and challenges. Nicotine Tob Res 2019;21:999–1000. 10.1093/ntr/ntz086 31112602

[10] MacKenzie R , Eckhardt J , Widyati Prastyani A . Japan Tobacco International: To 'be the most successful and respected tobacco company in the world'. Glob Public Health 2017;12:281–99. 10.1080/17441692.2016.1273368 28139966 PMC5553429

[11] Gilmore AB , Fooks G , Drope J , et al . Exposing and addressing tobacco industry conduct in low-income and middle-income countries. Lancet 2015;385:1029–43. 10.1016/S0140-6736(15)60312-9 25784350 PMC4382920

[12] Gilmore AB , Fooks G , McKee M . A review of the impacts of tobacco industry privatisation: implications for policy. Glob Public Health 2011;6:621–42. 10.1080/17441692.2011.595727 21790502 PMC3225958

[13] JT Becomes Majority Shareholder of Ethiopia’s NTE | Japan Tobacco International – a global tobacco company. Available: https://www.jti.com/jt-becomes-majority-shareholder-ethiopias-nte [Accessed 17 Jul 2020].

[14] Tobacco Reporter . JT group takes Ethiopian stake, 2016. Available: https://tobaccoreporter.com/2016/07/18/jt-group-takes-ethiopian-stake/

[15] Guliani H , Gamtessa S , Çule M . Factors affecting tobacco smoking in Ethiopia: evidence from the demographic and health surveys. BMC Public Health 2019;19:938. 10.1186/s12889-019-7200-8 31299938 PMC6624889

[16] Euromonitor International . Passport: tobacco in Ethiopia, 2020.

[17] Euromonitor International . Passport: tobacco in Ethiopia, 2019.

[18] Habebo TT , Takian A . Retrospective policy analysis of tobacco prevention and control in Ethiopia. Ethiop J Health Sci 2020;30:427–38. 10.4314/ejhs.v30i3.14 32874086 PMC7445944

[19] Erku DA , Tesfaye ET . Tobacco control and prevention efforts in Ethiopia pre- and post-ratification of who FCTC: current challenges and future directions. Tob Induc Dis 2019;17:13. 10.18332/tid/102286 31582924 PMC6751990

[20] Marquez PV . Ethiopia’s new tobacco control law: a step forward that needs to be complemented by higher taxes! 2019. Available: https://blogs.worldbank.org/health/ethiopia-s-new-tobacco-control-law-step-forward-needs-be-complemented-higher-taxes

[21] Federal Democratic Republic of Ethiopia . Food and medicine administration Proclamation No. 112/2019, 2019. Available: https://www.tobaccocontrollaws.org/files/live/Ethiopia/Ethiopia%20-%202019%20Proclamation%20-%20national.pdf

[22] Collin J . Tobacco control, global health policy and development: towards policy coherence in global governance. Tob Control 2012;21:274–80. 10.1136/tobaccocontrol-2011-050418 22345267 PMC4474152

[23] World Health Organization . Who framework convention on tobacco control guidelines for implementation of article 5.3, 2008. Available: http://www.who.int/fctc/guidelines/article_5_3.pdf

[24] Ethiopian Customs Commission . Memorandum of understanding between the Ethiopian customs Commission (CC) and the National tobacco enterprise (NTE, 2019.

[25] Greener I . The potential of path dependence in political studies. Politics 2005;25:62–72. 10.1111/j.1467-9256.2005.00230.x

[26] Federal Democratic Republic of Ethiopia . Minister of Council’s Implementation Directive Proclamation no. 1/1987, 2004.

[27] Lencucha R , Drope J , Labonté R , et al . Investment incentives and the implementation of the framework convention on tobacco control: evidence from Zambia. Tob Control 2016;25:483–7. 10.1136/tobaccocontrol-2015-052250 26135987 PMC4887414

[28] Lee S . What hinders implementation of the WHO FCTC Article 5.3? - The case of South Korea. Glob Public Health 2016;11:1109–20. 10.1080/17441692.2015.1122074 26708121

[29] Matthes BK , Robertson L , Gilmore AB . Needs of LMIC-based tobacco control advocates to counter tobacco industry policy interference: insights from semi-structured interviews. BMJ Open 2020;10:e044710. 10.1136/bmjopen-2020-044710 PMC7692838 33243822

[30] WHO . The tobacco industry and the illicit trade in tobacco products. Geneva: World Health Organization, 2014. https://www.who.int/fctc/publications/The_TI_and_the_Illicit_Trade_in_Tobacco_Products.pdf

[31] Crosbie E , Bialous S , Glantz SA . Memoranda of understanding: a tobacco industry strategy to undermine illicit tobacco trade policies. Tob Control 2019;28:e110–8. 10.1136/tobaccocontrol-2018-054668 30659106 PMC6639151

[32] Gilmore AB , Gallagher AWA , Rowell A . Tobacco industry's elaborate attempts to control a global track and trace system and fundamentally undermine the illicit trade protocol. Tob Control 2019;28:127–40. 10.1136/tobaccocontrol-2017-054191 29899082 PMC6580790

[33] Smith KE , Gilmore AB , Fooks G , et al . Tobacco industry attempts to undermine Article 5.3 and the "good governance" trap. Tob Control 2009;18:509–11. 10.1136/tc.2009.032300 19955541

[34] Smith KE , Fooks G , Collin J , et al . Is the increasing policy use of impact assessment in Europe likely to undermine efforts to achieve healthy public policy? J Epidemiol Community Health 2010;64:478–87. 10.1136/jech.2009.094300 20466716

[35] Smith KE , Fooks G , Gilmore AB , et al . Corporate coalitions and policy making in the European Union: how and why British American Tobacco promoted "Better Regulation". J Health Polit Policy Law 2015;40:325–72. 10.1215/03616878-2882231 25646389 PMC4668595

[36] Ndinda C , Ndhlovu TP , Juma P , et al . The evolution of non-communicable diseases policies in post-apartheid South Africa. BMC Public Health 2018;18:956. 10.1186/s12889-018-5832-8 30168397 PMC6117625

[37] World Bank . World bank group statement on current situation in Ethiopia, 2021. Available: https://www.worldbank.org/en/news/statement/2021/03/05/world-bank-group-statement-on-current-situation-in-ethiopia

[38] Del Biondo K . Democracy promotion meets development cooperation: the EU as a promoter of Democratic governance in sub-Saharan Africa. Eur Foreign Aff Rev 2011;16:659.

[39] Del Biondo K , Orbie J . The European Commission’s implementation of budget support and the Governance Incentive Tranche in Ethiopia: democracy promoter or developmental donor? Third World Q 2014;35:411–27. 10.1080/01436597.2014.893485

[40] Börzel TA , Hackenesch C . Small carrots, few sticks: EU good governance promotion in sub-Saharan Africa. Cambridge Review of International Affairs 2013;26:536–55. 10.1080/09557571.2013.807424

[41] Carbone M . The role of civil society in the Cotonou Agreement. In: Babarinde O , Faber G , eds. The European Union and the developing countries: the Cotonou agreement. Leiden: Martinus Nijhoff, 2005: 177–95. https://eprints.gla.ac.uk/49964/

[42] Rodwin MA . WHO’s Attempt to Navigate Commercial Influence and Conflicts of Interest in Nutrition Programs While Engaging With Non-State Actors: Reflections on WHO Guidance for Nation States Comment on "Towards Preventing and Managing Conflict of Interest in Nutrition Policy? An Analysis of Submissions to a Consultation on a Draft WHO Tool". Int J Health Policy Manag 2020;0. 10.34172/ijhpm.2020.162 PMC9278477 32979895

[43] UNDP . National tobacco control strategies: toolkit for parties to implement article 5.1 of the world Health organization framework convention on tobacco control, 2019. Available: https://www.undp.org/content/undp/en/home/librarypage/hiv-aids/national-tobacco-control-strategies.html [Accessed 23 Dec 2020].

